# Optimising α-Lactalbumin Recovery from Whey via Membrane Filtration: The Role of Transmembrane Pressure Across Membranes Varying in Polymer Type and Pore Size

**DOI:** 10.1007/s11947-026-04241-0

**Published:** 2026-02-16

**Authors:** Holly Giles, Alun Hughes, Joe Gallagher, Marianthi Faka, Stephanie P. Bull, Stella Lignou, Lisa Methven, David Warren-Walker

**Affiliations:** 1 Department of Food and Nutritional Sciences, Whiteknights University of Reading, Reading, RG6 6AP UK; 2 Institute of Biological, Environmental & Rural Sciences, Aberystwyth University, Plas Gogerddan, Aberystwyth, Ceredigion SY23 3EE UK; 3 Arla Foods Ingredients, Sønderhøj 10 -12, 8260 Viby J, Denmark

**Keywords:** Membrane filtration, Whey, Separation, Fractionation

## Abstract

There is strong commercial interest in the successful fractionation of whey protein into pure products. However, the effect of environmental conditions on membrane fractionation is not fully understood. In the current study filtration pressures, membranes of various pore sizes and types, and pH manipulation, were employed to investigate the effect of operating conditions on the enrichment of α-lactalbumin from whey protein. All of these factors were shown to affect the protein profile of the filtrate produced. Industrially relevant polyethersulfone microfiltration and ultrafiltration membranes, on a small-scale membrane filtration rig, were both able to filter out lactoferrin and lactoperoxidase, producing a filtrate that primarily contained β-lactoglobulin and α-lactalbumin. When scaled-up to a large cross-flow system, only the microfiltration membrane demonstrated a filtrate fractionation with an α-lactalbumin-enriched protein stream. When operated at 3 bar with a 1 in 3 dilution of a whey protein feedstock, a 140% increase in α-lactalbumin was achieved compared with a commercial whey protein liquid feed. This study shows the importance of the optimisation of all conditions during membrane fractionation and demonstrates that this technique can be used to produce an α-lactalbumin enriched product of industrial relevance.

**Supplementary Information:**

The online version contains supplementary material available at 10.1007/s11947-026-04241-0.

## Introduction

The disposal of whey represents a significant risk to the environment if not properly managed (Zandona et al. 2021), as well as a loss of potential nutrients and hence economic value. Therefore, there is environmental and commercial interest in the valorisation of whey to increase the sustainability of the dairy industry (Devine, 2021). To date, whey is utilised in concentrate and isolate forms in countless products, including fortified foods and beverages (Schmidt et al. 1984). Here, it provides nutritional benefits by increasing the protein content of foods, which is particularly relevant for oral nutritional supplements, both within the fitness industry and in products designed to address low protein intake (Cereda et al. 2022).

### The Composition of Whey Protein and the Need for Fractionation

Whey protein exists as a mixture of proteins including β-lactoglobulin, α-lactalbumin, glycomacropeptides, lactoferrin, lactoperoxidase, bovine serum albumin, and immunoglobulins (Etzel 2004; Madureira et al. 2007). The exact proportion of these components, as well as the presence of other non-protein compounds, depends on the level of processing. High purity versions of these proteins have been produced from *Saccharomyces cerevisiae *carrying bovine cDNA (Totsuka et al 1990), which has enabled initial investigations into the functions of these individual proteins. However, there is a need for further research to understand their individual physicochemical and sensory characteristics, ensuring that they are fully utilised to maximize their nutritional benefit.

Whilst the production of high purity proteins by bacteria is useful for analytical purposes, to address the consumer market it is necessary to optimise the commercial value of whey protein through enrichment of various protein fractions. Thus, a comprehensive, inexpensive and reliable method is required to enable enrichment of specific proteins from a whey protein feedstock. Membrane fractionation is a potential avenue to achieve this; to facilitate fractionation on a commercial scale, membrane filtration processes that are simple, rapid, and non-denaturing are employed, ensuring the production of a high-quality product (Chen et al. 2023).

### Previous Attempts to Fractionate Whey Protein Using Membranes

Due to the close size of α-lactalbumin and β-lactoglobulin (14 kDa and 18 kDa, respectively), most previous attempts to fractionate whey protein have collected these smaller proteins together. The earliest documented example of this was published by (Muller et al. 2003): the authors aimed to isolate a high purity α-lactalbumin, but were unable to efficiently remove β-lactoglobulin (Muller et al. 2003). (Almécija et al. 2007) investigated membrane fractionation under different conditions in order to separate these two proteins from whey and reported the highest yields for α-lactalbumin and β-lactoglobulin at pH 9.0, as 56% and 33% respectively, when using a 300 kDa tubular ceramic membrane (Almecija et al. 2007). Whilst this research has provided an interesting protein mixture of α-lactalbumin and β-lactoglobulin, there is considerable interest in the separation of α-lactalbumin and β-lactoglobulin to enable individual analysis and application of these proteins.

(Cheang and Zydney 2004)investigated the separation of β-lactoglobulin and α-lactalbumin from whey protein isolate using 100 kDa and 30 kDa regenerated cellulose membranes in series (Cheang and Zydney 2004), where a 90% yield of α-lactalbumin was reported. The authors state that the method has been optimised based on pH, buffer conductivity, and filtrate flux; however, the details of conditions investigated and the associated data are omitted. References to system pressure and temperature are also omitted, making this work difficult to replicate. Whilst this study provides promising results, it was conducted using laboratory-scale equipment and did not extend to industrial-scale application. Furthermore, the membranes employed were research-grade and are not currently industrially manufactured, limiting the direct applicability of these findings to industrial processes. In addition, the high yield reported in this study, does not align with the remaining literature, which reports significantly higher levels of retention of α-lactalbumin by ultrafiltration membranes (Almécija et al. 2007; Le Berre & Daufin, 1998). (Blais et al. 2022) state that “the wide range of partition behaviours reported of whey proteins in the literature, makes interpretation of likely rejection coefficients a complex task” (Blais et al. 2022).

In the literature, it has been reported that pre-treating materials may increase membrane selectivity and enable more effective separation of α-lactalbumin and β-lactoglobulin. pH manipulation is commonly used in ion exchange chromatography, but can be combined with membrane filtration due to the overlapping regions of the isoelectric points of proteins present in whey (Chen et al. 2023; Marciniak et al. 2020). Li et al.^2018^ induced aggregation of β-lactoglobulin through pH adjustment prior to membrane filtration to produce an α-lactalbumin-enriched whey protein concentrate (Li et al. 2018). Additionally, (Ye 2022) separated proteins based on differences in isoelectric point using dual-gating pH-responsive membranes, which, by sequentially increasing the pH, led to a pH-dependent change in membrane pore size (Ye et al. 2022). Whilst this was performed with a defined mixture of proteins present in whey, the individual proportions were not stated, meaning it is unclear if the mixture is representative of the proportions present in whey. If this mixture was composed of 25% of each protein, then the solutions’ properties are likely to vary significantly from those of whey protein isolate. In addition, the highly specific nature of the dual gated membrane fractionation system has not been reproduced to date, and would require extensive costings to be replicated in an industrial setting. However, it does provide substantial proof of concept for the individual fractionation of whey proteins using sequential changes in environmental conditions.

### Limitations in the Literature for Membrane Fractionation

Whilst promising, the existing literature demonstrates significant omissions which justify the need for further research: the majority of papers omit full details of the operating conditions used, limiting reproducibility. It is also noted that when this information is provided, it is rarely justified, meaning the impact of these choices on the experimental outcome is not understood. An example of this is the work of (Holland et al. 2012), which states that a range of temperatures and transmembrane pressures were tested in an optimisation process for milk; however, the results of these tests were not included, meaning the relationship between these conditions and the results cannot be elucidated (Holland et al. 2012). This highlights the need for a methodological investigation of membrane technology for this purpose, which will be addressed in this study.

Ultimately, there is a need to enhance the understanding of membrane fractionation methods to optimise the purity of fractions whilst minimising membrane fouling. Currently, membrane fouling is a major limiting factor against the industrial application of membrane filtration of whey protein due to high membrane costs and the frequent need for replacement (Wang et al. 2019). It is possible that increased knowledge of the effects of operating conditions would enhance protein separation efficiency and indirectly increase our understanding of fouling mechanisms to aid the uptake of filtration systems in the commercial processing of whey proteins.

### Aims and Hypotheses

To the authors’ knowledge, no comprehensive comparison has been undertaken comparing different membrane systems and operating conditions to separate individual proteins from a whey protein feedstock. Thus, there is an opportunity to increase understanding of the impact of these conditions, enabling the development of better membrane fractionation methodologies in the future. This study aims to compare the effects of different membrane sizes, membrane polymer material, operating conditions, and membrane filtration systems to understand the optimal conditions for fractionating an α-lactalbumin-enriched protein stream from a concentrated whey protein feedstock. A secondary aim of the study was to investigate the effect of commercial processing steps, including reverse osmosis and spray drying, on the properties of the product of membrane fractionation. The final aim was to generate a product which was food-grade and generated using methodologies that are compatible with standard product finishing techniques used across the industry.

## Methodology

An overview of the methodology has been provided in Supplementary Fig. 1: this schematic details the membrane filtration systems, operational parameters, and analysis techniques selected.

### Materials

A concentrated whey protein stream (WP) was provided in a liquid form by Volac Whey Nutrition Ltd (Hertfordshire, UK), immediately before spray drying. Industrially relevant polyethersulfone (PES) membranes were used, hereby referred to as microfiltration and ultrafiltration membranes to represent their respectively larger and smaller pore sizes. A polyacrylonitrile (PAN) ultrafiltration membrane with the same molecular weight cut off as the PES ultrafiltration membrane was also provided by the same supplier. Reverse osmosis was performed using RO-4040-FF membrane, supplied by FilmTec (Michigan, USA).

### Sample Preparation

When working on a small scale, 10% w/v suspensions were prepared by diluting WP with 18.2 MΩ-cm (ultrapure) Elga water and stirred using a magnetic stirrer for 60 min in a temperature-controlled room (20 °C). Suspensions were refrigerated overnight to ensure full homogenisation, and used within 24 h of production. To investigate the effect of pH, one batch was acidified by adding phosphoric acid to a pH of 3. All previous stirring protocols and timings were observed. All other materials were used without any manipulations to the pH.

When working at a large scale, 1 in 10 and 1 in 3 dilutions were prepared by diluting WP with potable water. The former of these was used to enable a direct comparison to the smaller scale system, whilst the 1 in 3 dilution was the highest concentration that could be used whilst maintaining an acceptable cross-flow. Stirring was not needed due to the recirculation stage provided by the membrane system facilitating shorted material handling times: when using this system the diluted materials were processed within 12 h of production.

### Dead-end Pressure Cells (Small-Scale Work)

A HPC001 high-pressure stirred cell (Membranology Ltd, Swansea, UK) with a total volume capacity of 300 mL, a maximum operational pressure allowance of 100 bar, and a membrane area of 50.27 cm2 was used for small-scale membrane fractionations. The system was pressurised with nitrogen gas and controlled via valves and digital pressure gauges. This system has been previously described in the literature (Gerardo et al. 2015; Oatley-Radcliffe et al. 2015) and the system flow visualised in Supplementary Fig. 2 (SF2). The cell was operated at pressures of 2, 4, 6, and 8 bar, with a stirrer set at a constant speed (setting 4, ARE Magnetic Stirrer) and the temperature monitored (18–25.6 ℃). Membranes were cut to size and preconditioned by immersing overnight in deionised water or ethanol, as appropriate, and refrigerated for a minimum of 12 h before use. Before introducing the whey protein samples, each membrane was flushed with deionised water at 6 bar. Membranes were cleaned with 100 mM sodium hydroxide between each sample. During membrane fractionation, 300 mL of 10% w/v WP suspension was added to the pressure cell. Sequential collection of 20 mL of filtrate was completed until a total of 240 mL of filtrate had been collected. The remaining 60 mL retentate, with a concentration factor of 5, was collected and analysed.

### Cross Flow Membrane Fractionation Rig (Large-Scale Work)

The large-scale membrane fraction rig was manufactured by Axium (Swansea, UK) and utilises a 3838 housing with a maximum pressure of 3.5 bar. The reverse osmosis rig, also by Axium, has a 4040 housing with a maximum pressure of 68 bar. The rig was operated at 1, 2, 3 and 3.4 bar applied pressure using two Sanitary Ultrafiltration Spiral-Wound Element membrane cassettes. During investigative membrane fractionation, 100 L of 10% w/v WP suspension was added to the tank and recirculated. The temperature of the filtrate was monitored (6.4–13.6 ℃). 50 mL of filtrate was collected in 5-min intervals and analysed alongside the start and retentate material. During the production run, completed at 3 bar, 500 L of 33% w/v WP suspensions was added using an external tank and recirculated. The temperature of the filtrate was monitored (6.5–19.6 ℃). This system has been visualised in Supplementary Fig. 3 (SF3) for additional clarity.

### Reverse Osmosis

A reverse osmosis approach was employed to increase the concentration of dissolved solids in the permeate produced during cross-flow membrane fractionation, thereby facilitating more efficient spray drying. The material was recirculated in a 500-L refrigerated dairy missing tank, at an inlet pressure of 25 bar and an outlet pressure of 24 bar until the retentate volume was below 100 L, at which point the retentate was recirculated within the RO system’s in-built tank. The RO was stopped when either the Brix reading achieved the same value as the original WP concentration or the system’s dead volume was reached (~14 L).

### Spray Drying

RO concentrated membrane fractionated WP samples were spray dried to produce a fine dry powder. Spray drying was conducted using an Armfield FT80 tall form spray drier. Feedstock was pumped into the top of the drying tower through two fluid nozzles, where the feed passed down a central bore with increasing constriction and was surrounded by compressed air at 750 mbar, resulting in the atomisation of the feed sample. Air is pumped in (the inlet fan is set to 28 Hz, and the inlet temperature is set to 190 °C), which surrounds the nozzle and carries the atomised feedstock down the column. The drying time in the tower is approximately 9 s. The outlet fan speed was set between 34 and 36 Hz, and the speed of the feed pump varied to achieve a target relative humidity of 12.0%. Pilot work has highlighted these parameters as optimal for WP to generate a powder with a moisture content of 4–5% (unpublished).

### Analysis of Total Solids and Proteins Collected Through Membrane Fractionation

#### Brix Measurements

Samples were collected for Brix measurements as an estimation of the total solid content using a RSA-BR82T refractometer, supplied by Cole-Parmer (Cambridgeshire, UK). This was completed using 500 μL of filtrate measured immediately after collection. Deionised water was used to clean the equipment until a measurement of 0 ⁰Bx was obtained when reading a sample of deionised water. Readings were taken by two independent researchers to ensure consistency.

#### SDS-PAGE

To enable a qualitative estimation of the protein profile of membrane fractions alongside the protein processing, sodium dodecyl sulphate-polyacrylamide gel electrophoresis (SDS-PAGE) was used. Samples were prepared for sodium dodecyl sulphate-polyacrylamide gel electrophoresis (SDS-PAGE) by combining with a reducing 10% SDS buffer in a ratio of 2:1. This was heated at 85 ℃ for 9 min to be denatured. An aliquot (10 μL) of each sample was loaded onto the gels, alongside 3 μL of a pre-stained broad-range protein ladder (New England BioLabs, Ipswich, UK). Samples were then loaded on a Tris-acrylamide gel (Bio-Rad, Ontario, Canada; 12% Mini-PROTEAN TGC Precast Protein Gels, 10 wells). SDS-PAGE was performed using a Bio-Rad Mini-PROTEAN electrophoresis kit (Ontario, Canada) at 100 mV in buffer (3.03 g Trizma base, 14.4 g glycine, 1 g SDS per litre) until the proteins were observed <1 cm from the bottom of the gel (usually 80–90 min). After running the gels were: stained with Coomassie blue stain (25% w/v Coomassie blue, 50% methanol, 40% water and 10% glacial acetic acid per litre) for a minimum of 1 h; destained (ethanol (30%), acetic acid (10%) and water (60%)) for a minimum of 1 h or until visually acceptable; and digitally imaged.

#### Freeze Drying and Mass Balance

Collected subsamples from membrane permeate and retentate taken throughout the process were frozen at −20°C overnight, before being freeze-dried for 72 h (Edwards, Freeze Dryer Super Modulyo). Samples were weighed both before and after freeze drying to calculate the mass of solids for mass balance calculations.

#### Characterisation of Protein Profiles

For final quantitation of the protein profile of both starting material and products, HPLC methods were carried out by NIZO Food Research BV (Ede, Netherlands). This followed a commercially validated method for each protein: caseinomacropeptide (CMP), α-lactalbumin, and β-lactoglobulin were analysed with Reverse-Phase High Pressure Liquid Chromatography and UV (RP-HPLC-UV); IgA, IgG, and BSA were determined by High Performance Size Exclusion Chromatography with Ultra Violet (HP-SEC-UV) detection; and Reversed Phase High Pressure Liquid Chromatography and UV (RP-HPLC-UV) was used for the quantitative analysis of lactoferrin. A pre-treatment of the samples was performed to isolate and concentrate the lactoferrin.

## Results and Discussion

### Characterisation and Manipulation of the WP Feedstock

**Fig. 1:**
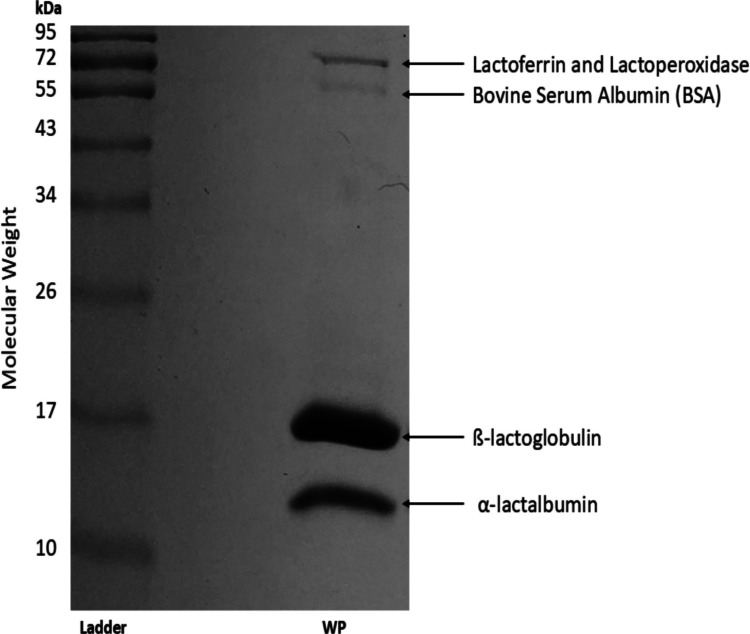
SDS PAGE Protein profile of 10% (v/v) aqueous solution of a concentrated whey protein feedstock. 13 µg protein loaded per lane in a 2:1 ratio with sample buffer

When characterising a 10% w/v WP suspension using SDS-PAGE (following methodology detailed in the “SDS-PAGE” section), four protein bands were seen; each corresponding to the size of a protein fraction present within WP (Fig. 1). It is noted that the intensity of the β-lactoglobulin and α-lactalbumin bands are much higher than those of lactoferrin, lactoperoxidase and BSA due to their higher estimated proportion of the protein present in WP (Table 1). This is consistent with the literature (Etzel 2004; Madureira et al. 2007)*.*It is known that a proportion of β-lactoglobulin exists in a dimerised form within whey protein (Mercadante et al. 2012); however, this was not observed in the gel as the dimer is likely to be broken during the denaturation process (Nowakowski et al. 2014). The presence of a dimer underscores why the transmission of proteins through membrane filtration cannot be assumed based solely on size, as this approach overlooks protein–protein interactions. This characterisation of the starting feedstock serves as a reference point for the remainder of the study, enabling an understanding of how membrane filtration has influenced the protein profile of the material. This concentration (10% w/v) was the highest concentration which could be used, whilst maintaining an acceptable filtration rate and minimal fouling: higher concentration solutions demonstrated excessive viscosity for filtration using the dead-end filtration system.

**Table 1:** Characteristics and proportions of key proteins within a concentrated whey protein (WP) feedstock. Adapted from (Etzel 2004) with additional information from (Madureira et al. 2007)

Protein	Estimated proportion within WP (%)	Isoelectric point	Molecular weight (kDa)
Immunoglobulins	8–12	5.5–8.3	80–900
Lactoferrin	2	8.4–9.0	80
Lactoperoxidase	0.5	9.6	78
Bovine serum albumin	6	4.7–4.9	66
Glycomacropeptide	12–20	< 3.8	14–30
Β-lactoglobulin	48–58	5.4	18
α-lactalbumin	13–19	4.2–4.5	14

The isoelectric point is different for each of the proteins present within whey (Table 1). Previous literature has highlighted the opportunity of pH manipulation to separate proteins (Chen et al. 2023; Ye et al. 2022), thus the effect of reducing the pH to below that of all of the individual protein isoelectric points was investigated, by comparing the native pH of the whey protein material (pH 6.5), with an acidified solution (pH 3.2). The effect of lowering the pH was shown to have a negative impact on membrane fractionation (Supplementary Fig. 4), which was hypothesised to be a result of increased interactions between particles, leading to increased aggregation. This is likely to explain the inability of proteins to pass through the membrane pores as the interactions would influence their size and charge. It is also anticipated that a change in pH will alter the charge on the membrane (Kimani et al. 2022). An increase in interactions as a response to acidification was previously discussed by Chen et al. (2023). In addition, (Cheang and Zydney 2004)reported poor selectivity and considerable fouling when performing membrane filtration under acidic conditions (Cheang et al. 2004). Their paper stated an optimisation process based on pH but the final working pH was omitted, limiting comparisons that can be made. Whilst it was outside the scope of the current study to investigate pH further, this highlights the impact of pH on the behaviour of WPI during filtration and the need to report the pH of materials. It justifies the selection of a neutral pH for the remainder of the study as this showed a better filtration profile (Supplementary Fig. 4).

### Selection of Membrane Material and Interactions with the Whey Protein

**Fig. 2:**
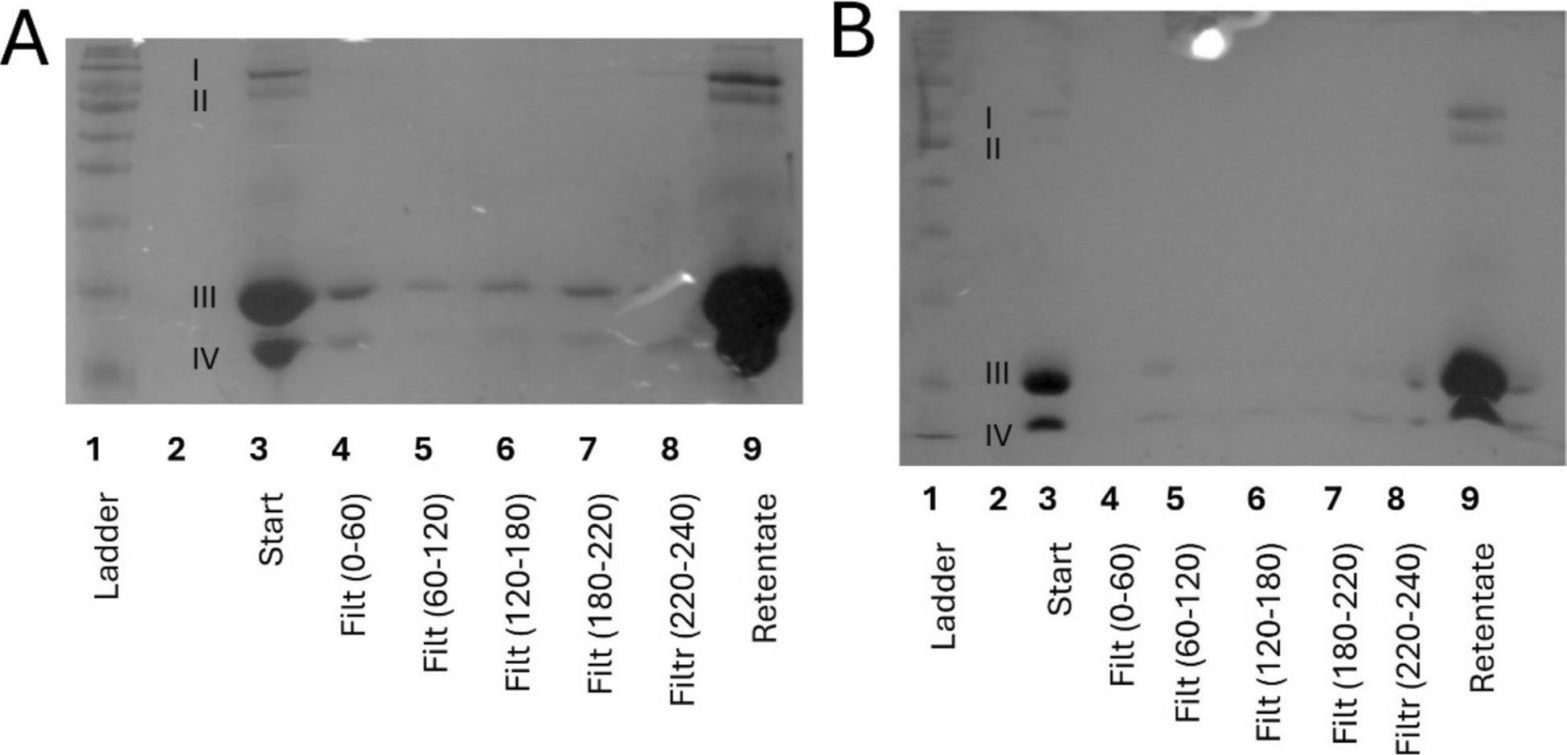
SDS-PAGE (following methodology detailed in the “SDS-PAGE” section) of filtrates of 10% w/v whey protein suspension at 8 bar using an industrially relevant ultrafiltration membrane with different backbones: (**A**) polyethersulfone membrane; (**B**) polyacrylonitrile membrane. Each well represents subsequent collections of filtrate with the millilitre reference included in brackets, which have been compared with a protein ladder, the starting solution, and the retentate. Bands labelled I–IV correspond with the associated size of: (I) lactoferrin and lactoperoxidase; (II) bovine serum albumin; (III) β-lactoglobulin; (IV) α-lactalbumin. Equal amounts loaded into each well (e.g., not normalised by protein content), retentate has a concentration factor 5

To understand the effect of different membrane polymer materials, the filtrates from fractionation using two ultrafiltration membranes made from polyethersulfone (PES) and polyacrylonitrile (PAN) were compared using SDS-PAGE (following methodology detailed in the “SDS-PAGE” section). PES is reported to have high strength, creep, temperature and chemical resistance (Kerr-Phillips et al. 2022). In addition, PES is less hydrophilic than PAN which is likely to cause it to interact differently with charged proteins (Badru et al. 2024). When comparing the filtrates produced with the two membranes operating at 8 bar, it was demonstrated that the PES membrane allowed α-lactalbumin and β-lactoglobulin to pass through. In contrast, the PAN membrane did not show substantial levels of any protein in the filtrate (Fig.2). It is possible that the less hydrophilic nature of the PES membrane led to increased interactions with the whey protein, allowing them to more easily pass through the membrane. This highlights the importance of membrane selection and the need for further research in this area. A wide range of membrane materials are cited in the literature (the “Previous Attempts to Fractionate Whey Protein using Membranes” section). However, the authors are not aware of any paper that justifies their selection with experimental data. The results of the current study demonstrate the importance of the membrane polymer material, and the necessity for more transparent reporting in the literature, as well as the need for future comparative research in this area.

### Small-Scale Work Highlighting the Impact of Operating Conditions

**Fig. 3:**
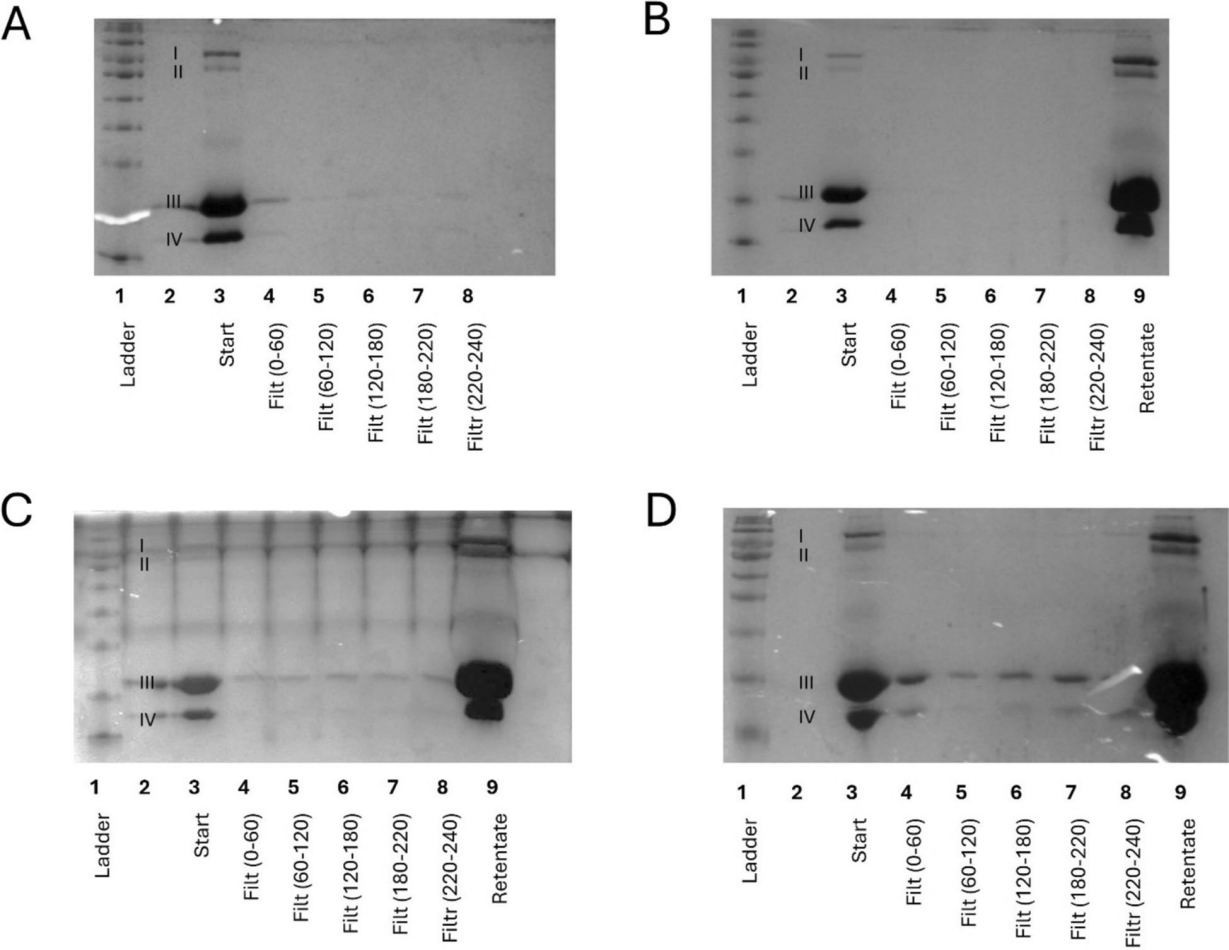
SDS-PAGE of filtrates (filt) produced through membrane fractionation of 10% w/v whey protein solution using an industrially relevant ultrafiltration membrane using a small-scale dead one membrane filtration rig at different pressures: (**A**) 2 bar; (**B**) 4 bar; (**C**) 6 bar; (**D**) 8 bar. Each well represents subsequent collections of filtrate with the millilitre reference included in brackets, which have been compared with a protein ladder, the starting solution, and the retentate. Bands labelled I–IV correspond with the associated size of: (I) lactoferrin and lactoperoxidase; (II) bovine serum albumin; (III) β-lactoglobulin; (IV) α-lactalbumin. Equal amounts were loaded into each well (e.g., not normalised by protein content), retentate has a concentration factor of 5

To address the omissions of previous literature, small-scale dead-end filtration systems were employed to investigate the impact of operating conditions on filtration. The effect of a range of pressures (2, 4, 6, and 8 bar) on protein separation with an ultrafiltration membrane was investigated qualitatively using SDS-PAGE (Fig. 3), following methodology detailed in the “SDS-PAGE” section. When filtering 10% w/v whey protein suspensions at 2 and 4 bar, minimal amounts of protein were observed in the filtrates (Fig. 3A and B); this pressure was insufficient to facilitate the passage of protein through the membrane. However, at 6 and 8 bar, an increasing presence of bands correlating to the sizes of α-lactalbumin and β-lactoglobulin were observed in the filtrate (Fig. 3C and D). Bands for lactoferrin and lactoperoxidase were not observed in the filtrates at any pressure, suggesting that these proteins remained in the retentate; this was confirmed using filtrate samples concentrated fourfold, where no lactoferrin or lactoperoxidase corresponding bands were observed (data not shown). The assessment of pressure using an ultrafiltration membrane demonstrates that it is possible to fractionate lactoferrin and lactoperoxidase from WP, but that it is dependent on sufficient pressure.

At 6 and 8 bar pressure, the filtrate contained both β-lactoglobulin and α-lactalbumin. However, within the 6–8 bar filtrates, the proportions of each of these proteins was different as indicated by the intensity of the bands (Fig. 3C and D). The implication from this research is that a UF membrane can fractionate α-lactalbumin and β-lactoglobulin from lactoferrin and lactoperoxidase with an enrichment of the β-lactoglobulin protein (band III) within the filtrate. During the fractionation process, pH was shown to change by ≤ 0.50 and, hence, did not cross the boundary of the isoelectric point of any of the proteins present (Table 1), and as such was not anticipated to have impacted the filtration results. This was consistent across all membrane fractionation parameters tested. It is also noted that the concentration factor of the retentate was 5, meaning that this band is significantly brighter on the gels; however, this does not impact interpretation of results which focus on the filtrate lanes (lanes 4–8).

**Fig. 4:**
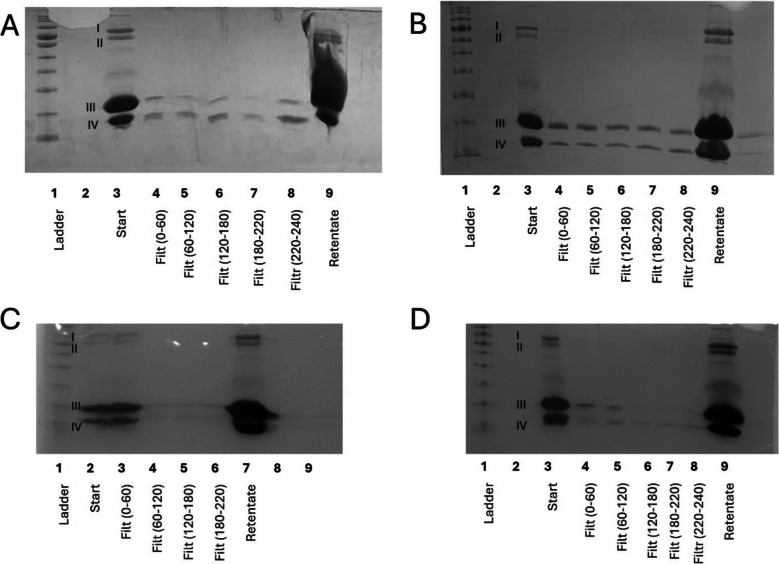
SDS-PAGE of filtrates (filt) produced through membrane fractionation of 10% w/v whey protein solution using an industrially relevant polyethersulfone microfiltration membrane using a small-scale dead one membrane filtration rig at different pressures: (**A**) 2 bar; (**B**) 4 bar; (**C**) 6 bar; (**D**) 8 bar. Each well represents subsequent collections of filtrate with the millilitre reference included in brackets, which have been compared with a protein ladder (L), the starting solution (St), and the retentate (R). Bands labelled I–IV correspond with the associated size of: (I) lactoferrin and lactoperoxidase; (II) bovine serum albumin; (III) β-lactoglobulin; (IV) α-lactalbumin. Equal amounts were loaded into each well (e.g., not normalised by protein content), retentate has a concentration factor of 5

To assess the effect of pore size, a microfiltration PES membrane was used which has a larger pore size than the molecular weight of all whey proteins. The use of such a membrane is perhaps counter-intuitive as it might be anticipated that it would not lead to protein separation. However, as previously discussed, proteins can self-associate in solution, leading to complex interactions (including dimerization) (Madrureira et al. 2007; Mercadante et al. 2012) and, therefore, the molecular weight of the proteins are not a reliable indicator of how they might respond to a given pore size. The microfiltration membrane resulted in filtrates that were enriched with α-lactalbumin, leading to similar levels (at least qualitatively) of α-lactalbumin and β-lactoglobulin (Fig.4).

In contrast to the previous results, the microfiltration membrane demonstrated a negative correlation between filtrate protein concentration and pressure (Fig. 4): the most intense bands in the filtrate were recorded at 2 and 4 bar (Fig. 4A and B), with a reduction in band intensity observed as pressure increased (Fig. 4C and D). This may indicate a real filtration effect, suggesting that the ultrafiltration membrane, in contrast, was demonstrating force-through of proteins at a higher pressure regardless of size. Dead-end membrane systems have been observed to have lower fluxes and rejection selectivity that true cross flow systems using the same membrane chemistry (Shamsuddin et al. 2015), highlighting the need for true cross-flow membrane systems for investigative purposes. Whilst providing good proof of principle, it is also noted that dead-end systems are more variable, as differences in stirrer shape and distance from the membrane influence mixing efficiency and filtration (Imbrogno & Schäfer, 2019): a cross-flow mechanism can be more readily standardised, which is essential for comparable results and industry applications.

### Large-Scale Fractionation and the Transferability of Fractionation Conditions

To address the limitations inherited with small-scale membrane rigs, a true cross-flow filtration system was used to further investigate the effect of pressure on fractionation. The initial target was to replicate the pressure sweep undertaken at laboratory scale, thereby validating the performance of each membrane at pressures of 2, 4, 6, and 8 bar. This was not possible, however, due to the pressure limitations of the pilot-scale filtration system used in this study, which cannot exceed 3.4 bar. A screen at lower operational pressures was used, at 1, 2, 3 and 3.4 bar, to investigate the combined effects of increased trans-membrane pressure and reducing cross flow rate on the flux and selectivity of the separation process. In the future, it would be beneficial to investigate the operation of these membranes on a pilot-scale system capable of operating at elevated pressures to further understand the relationship between pressure and filtrate profiles.

**Fig. 5:**
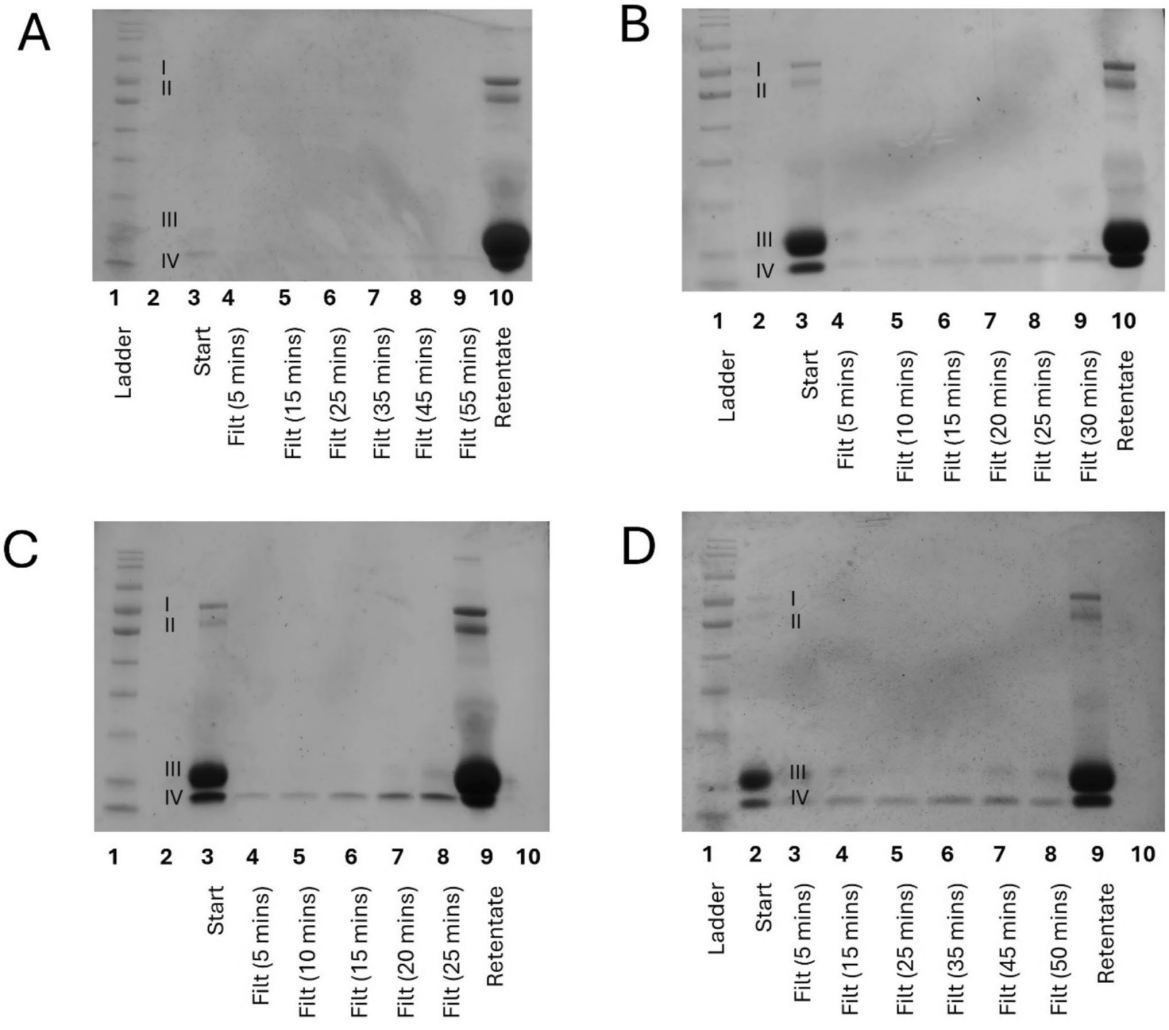
SDS-PAGE of filtrates (filt) produced through membrane fractionation of 10% w/v whey protein solution using industrially relevant polyethersulfone microfiltration membrane on a large-scale cross-flow membrane filtration rig collected every 5 min at different pressures: (**A**) 1 bar; (**B**) 2 bar; (**C**) 3 bar; (**D**) 3.4 bar. Equal amounts were loaded into each well (e.g., not normalised by protein content), concentration factors of retentate: (**A**) 2.2; (**B**) 4.76; (**C**) 5.3; (**D**) 3.8. Bands labelled I–IV correspond with the associated size of: (I) lactoferrin and lactoperoxidase; (II) bovine serum albumin; (III) β-lactoglobulin; (IV) α-lactalbumin

A microfiltration membrane was used to fractionate a 1 in 10 dilution of whey protein: this concentration enabled a direct comparison with the dead-end filtration system. When using the microfiltration membrane, a positive relationship between protein concentration in the filtrates was seen with increasing pressure (Fig. 5), when assessed qualitatively using SDS-PAGE. This was consistent with observations made at the small-scale. It was also observed that in this large-scale membrane system the protein profile of the filtrates was almost exclusively composed of α-lactalbumin (band IV, Fig. 5), with very minimal levels of β-lactoglobulin (band III, Fig. 5). Literature using different feedstocks also reports better efficiency and membrane selectivity in a circular flow system compared with a dead-end pressure cell (Shamsuddin et al. 2015; Wang et al. 2022). It is likely that the nature of the cross-flow system and the ability to recirculate material using this rig facilitated the same effect as the dead-end system, but at a lower pressure. The differing filtrate volumes collected mean that the retentate has different concentration factors (A – 2.2, B – 4.76, C – 5.3, D – 3.8, Fig.5); however, this this does not affect the ability to draw comparisons between the filtrates of the four operational conditions.

**Fig. 6:**
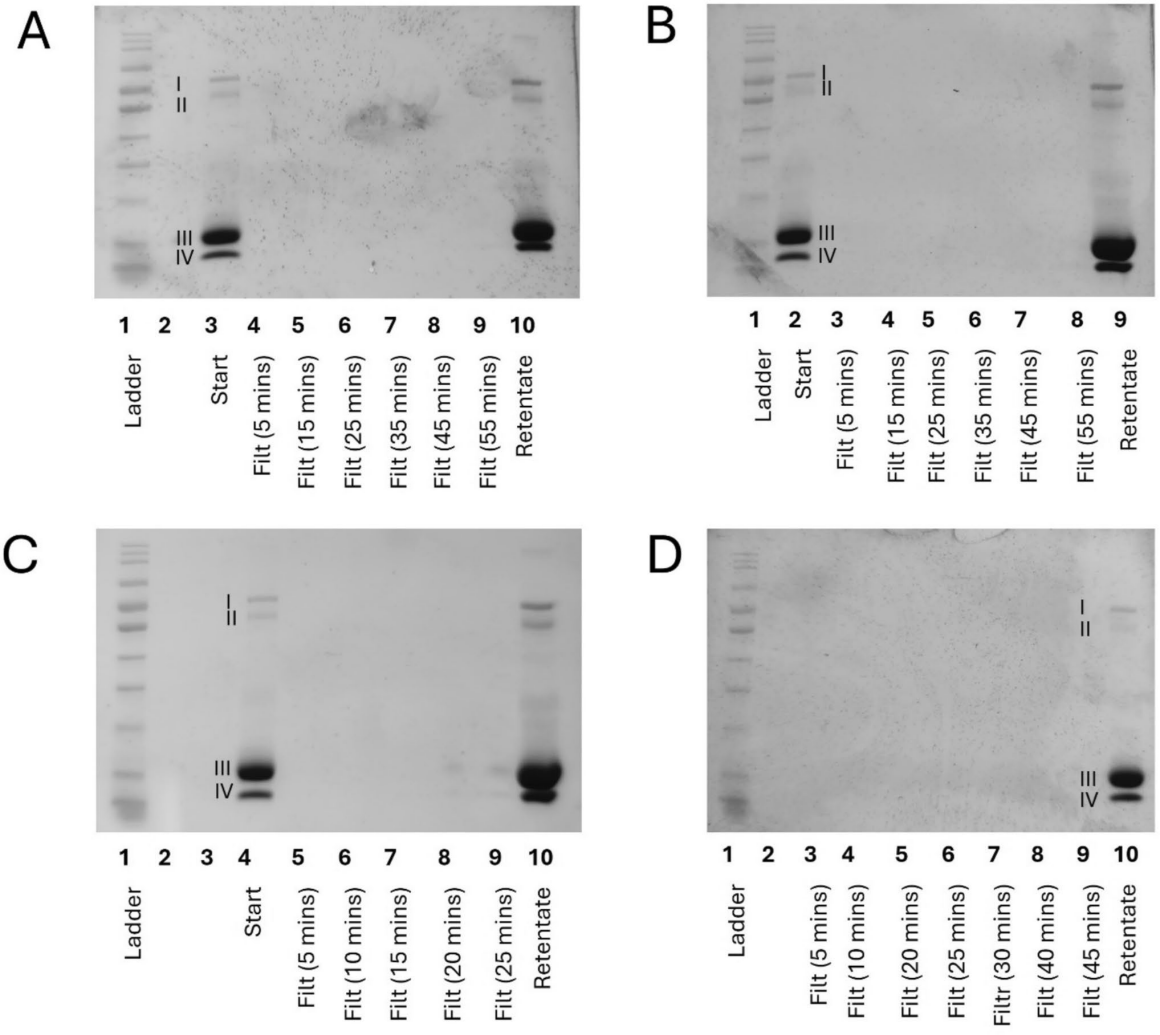
SDS-PAGE of filtrates (filt) produced through membrane fractionation of 10% w/v whey protein solution using industrially relevant polyethersulfone ultrafiltration membrane on a large-scale cross-flow membrane filtration rig collected every 5 min at different pressures: (**A**) 1 bar; (**B**) 2 bar; (**C**) 3 bar; (**D**) 3.4 bar. Bands labelled I–IV correspond with the associated size of: (I) lactoferrin and lactoperoxidase; (II) bovine serum albumin; (III) β-lactoglobulin; (IV) α-lactalbumin. Equal amounts were loaded into each well (e.g., not normalised by protein content), concentration factors of retentate: (**A**) 2.3; (**B**) 4.29; (**C**) 4.83; (**D**) 4.03

Despite a difference in the relationship with pressure, good transferability was observed between the two systems when using a microfiltration membrane. However, when using the ultrafiltration membrane on the cross-flow system, no fractionation was observed, irrespective of pressure (Fig. 6). This supports the previous suggestion that the earlier results from the ultrafiltration membrane were due to force-through, rather than an actual fractionation effect.

### Assessment of Protein Yield Through Fractionation Methods

**Fig. 7:**
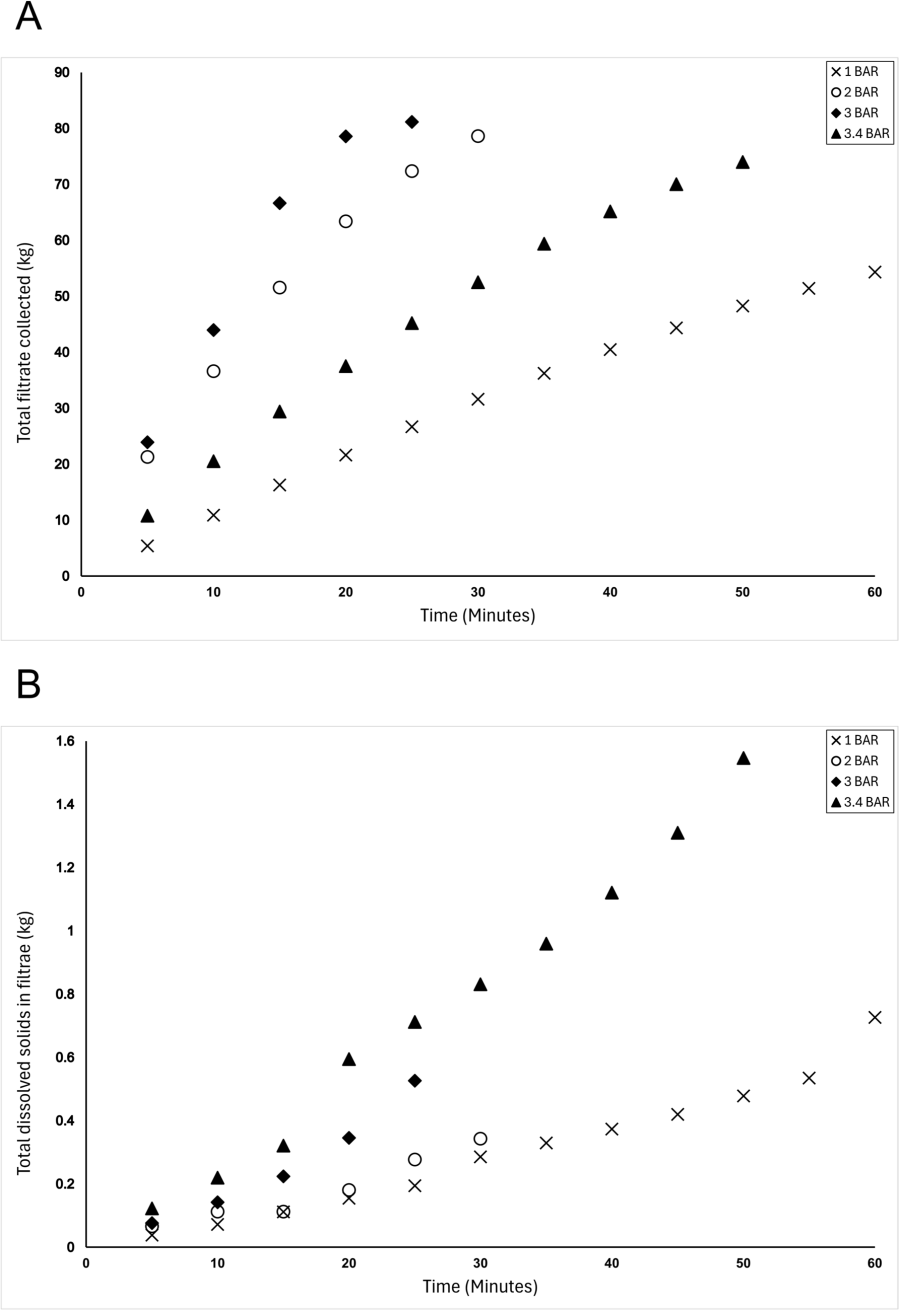

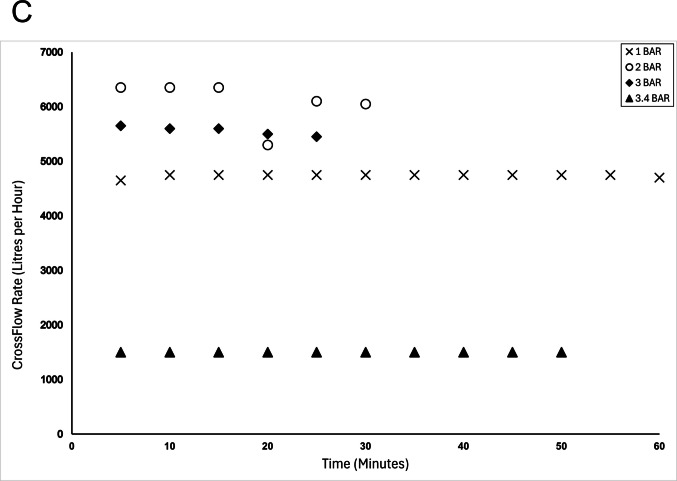
Analysis of the filtrates produced when using industrially relevant polyethersulfone microfiltration membrane to fractionate 10% w/v whey protein feedstock: (**A**) Filtrate flow rate (kg/min) at different pressures; (**B**) Mass of protein produced per 50 mL of filtrate every 5 min at different pressures; (**C**) Cross flow rate (L/hour) every 5 min at different pressures

In order to apply such filtration technologies at an industrial scale, it would be essential to calculate protein yield in the filtrates. Within this study, the initial approach was to use SDS-PAGE to provide a qualitative assessment of the protein profile of the filtrates: this enables visualisation of the proteins present and comparisons between similar runs, but cannot assess the overall protein yield. Ideally, a simple total protein method, such as a colorimetric method, that could be used in real-time with the membrane processing would have been chosen. However, the Bradford assay, the most common rapid protein determination method, has been previously shown to be inappropriate in this context due to the different binding affinities of each protein fraction (Giles et al. 2026). Therefore, to give an estimate of protein yield in the filtrates, two approaches were taken; measurement of total solids (estimated by Brix), and calculation of mass balance. As the starting material primarily consists of only protein and water, this enables these two approaches to be used. The mass data was generated by freeze-drying sub-samples of the filtrates: this suggested that increased protein concentrations in the filtrates were associated with better yield, allowing this measure to be used as an estimate of protein content (data not published). By measuring the Brix content of the filtrate, it was shown that increased pressure was associated with an increase in mass of protein being collected in the filtrate (Fig.7B), suggesting that 3.4 bar was the optimal pressure of those tested for fractionation. This is consistent with the SDS-PAGE gels, where more intense bands are observed in the filtrate at 3 and 3.4 bar (Fig. 6C and D).

Optimal conditions will demonstrate a balance between a reasonable membrane crossflow rate and a high yield of protein. This was investigated by recording the amount of filtrate produced (Fig. 7A) and the cross flow (Fig. 7C). Whilst 3.4 bar yielded the highest levels of dissolved solids within the filtrate (Fig. 7B), the crossflow rate was unacceptably low, leading to concerns regarding membrane fouling and force through. This would have commercial implications and reduce the sustainability and costs associated with membrane fractionation at an industrial scale. Thus, it was determined that 3 bar was the most suitable pressure for an industrial membrane system of the investigated options using the current membrane system.

### Optimisation of an Enriched α-Lactalbumin Stream Production

**Fig. 8:**
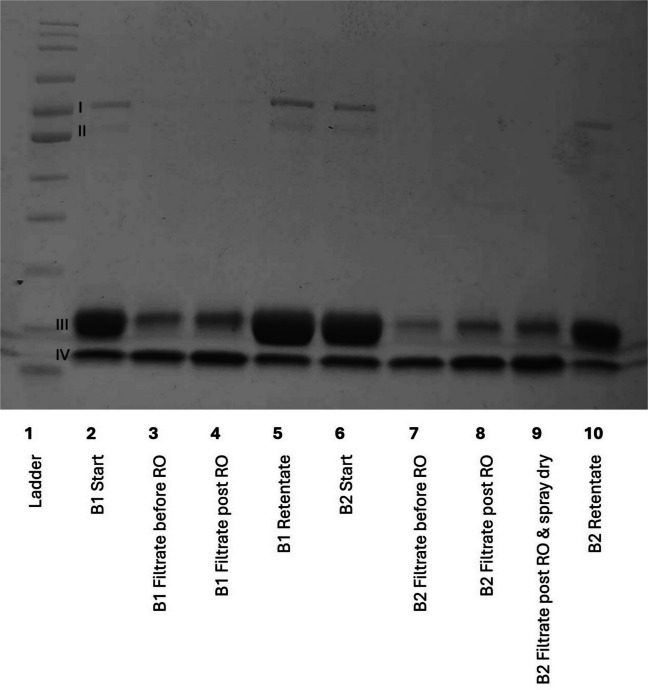
SDS-PAGE of membrane filtration of 33% w/v whey protein solution using industrially relevant polyethersulfone microfiltration membrane on a large-scale cross-flow membrane filtration rig at 3 bar. Batch 1 (B1) completed with minimal diafiltration, and Batch 2 (B2) completed with extensive diafiltration to increase yield. Bands labelled I–IV correspond with the associated size of: (I) lactoferrin and lactoperoxidase; (II) bovine serum albumin; (III) β-lactoglobulin; (IV) α-lactalbumin. Normalised loading based on Brix content across the gel

A 10% w/v suspension was used for all work on the dead-end pressure cell, due to the formation of a stagnant film boundary layer over the membrane (Imbrogno & Schäfer, 2019). However, preliminary investigations suggested that the large-scale pressure rig enabled the use of higher concentrations of feed material whilst maintaining an acceptable cross-flow: to increase the protein concentration in the filtrates, and the ease of subsequent spray drying, a 1 in 3 dilution was used as the feed material when working with this system (100 kg of WP feed material, diluted with 200 L of potable water). This was the highest concentration that could be used whilst maintaining acceptable cross-flow characteristics and flux, so was used as the most efficient for membrane fractionation on this system. Throughout the process, the permeate flow rate, retentate cross-flow rate and transmembrane pressure were measured and recorded. As the volume of recirculated retentate decreased, non-membrane permeable molecules such as lactoferrin and lactoperoxidase were concentrated, increasing the viscosity of this stream, which caused a reduction in the cross-flow rate. To maintain an acceptable cross-flow rate and maximise fractionation of the four key proteins into the two streams, a diafiltration approach was adopted. In the first batch (B1 in Fig.8), minimal diafiltration was performed with 30L being added during the filtration process, whereas in the second batch (B2 in Fig. 8), 400 L of potable water was used to dialyse the solutions, yielding 500 L of permeate at 2° Brix. When comparing these two batches, it was observed that diafiltration increased the purity of the enriched α-lactalbumin stream, as indicated by the increased intensity of the α-lactalbumin band and the decreased intensity of the β-lactoglobulin band (lanes 3 and 7, respectively, Fig. 8). This is matched by a decrease in the amount of α-lactalbumin remaining in the retentate of batch 2 compared with batch 1 (lanes 5 and 10, respectively, Fig. 8), supporting the conclusion that diafiltration increased the amount of α-lactalbumin in the filtrate. This is in line with previous research, where diafiltration enables the concentration of peptides and immunoglobulins in the permeate, with an enriched retentate of β-lactoglobulin and α-lactalbumin (Xu et al. 2000). It was also demonstrated that the use of reverse osmosis to increase the concentration of the filtrate solution had no effect on the protein profile (lanes 7–8, Fig.8). This is of commercial relevance with regard to scale-up and utilisation of this method.

### Characterisation of Membrane Fractionation Products

Spray-dried samples of the membrane fractionation products were assessed quantitatively for both total protein, and for each specific protein, using the appropriate HPLC methods (the “Characterisation of Protein Profiles” section). The total protein content was comparable between the filtrate, retentate and start material (data not shown), suggesting that protein had not been lost during membrane fractionation. The proportion of each protein present in the sample was calculated using protein profiling. Here, it was shown that 53% of the protein present in the filtrate was α-lactalbumin, compared with 22% in the WP starting material: membrane fractionation had increased the proportion of α-lactalbumin present in the sample by 140%. By contrast, the retentate contained 19% α-lactalbumin and 67% β-lactoglobulin, showing a clear difference in protein profiles between the two products (Table 2). Limited research exists on the physicochemical and sensory properties of these proteins, meaning that the profiles of the retentate and filtrate cannot be concluded in the current study, but this is an essential avenue for future research.

**Table 2:** Percentage of protein present in whey protein starting material, filtrate and retentate in the freeze dried samples after membrane fractionation with an industrially relevant polyethersulfone microfiltration membrane. Estimates provided for glycomacropeptides (GMP), α-lactalbumin (ALA), β-lactoglobulin (β-LG), immunoglobulins (IgA and IgG), bovine serum albumin (BSA) and lactoferrin (LF) in the freeze dried samples. Proteins quantified by NIZO Food Research BV (Ede, Netherlands) through HLPC, as detailed in the **“**Characterisation of Protein Profiles” section

Sample	% GMP	% a-LA	% b-LG	%IgA	% IgG	% BSA	% LF
Start	14.2	22.7	59.7	0.2	1.6	1.6	0.03
Filtrate	37.0	53.2	9.7	0	0	0.1	0
Retentate	11.4	19.0	66.6	0.2	1.3	1.4	0.03

## Conclusion

The commercial interest in the successful fractionation of whey protein into isolated protein components means much of the existing literature omits key methodological details. This study investigated the effect of operating conditions, membrane types, and membrane systems, on filtrate production. We have shown that these factors all have a large effect on the protein profile of the filtrate produced, and as such, should be reported in all future literature. The authors suggest that increased transparency in reporting will propel the advancement of this field.

When using a small-scale membrane filtration system, it was shown that both ultrafiltration and microfiltration membranes were able to filter out lactoferrin and lactoperoxidase, producing a filtrate that primarily contained β-lactoglobulin and α-lactalbumin. However, the relative proportions of these two components varied with membrane type. When up-scaled to an industrially relevant pilot scale cross-flow filtration system, it was shown that the ultrafiltration membrane did not allow any notable amount of protein to permeate, suggesting that a force-through effect had been observed in the small-scale system. Contrastingly, the microfiltration membrane demonstrated good separation and the ability to produce an α-lactalbumin-enriched protein stream when operating at 3 bar with a 1 in 3 dilution of a whey protein feedstock: the final permeate consisted of 53% α-lactalbumin, 37% GMP and 9% β-lactoglobulin. This is an increase in α-lactalbumin levels by 140% compared with a commercial whey protein, which could have significant application potential. We have demonstrated a clear methodology for creating an α-lactalbumin-enriched protein product from WP using membrane fractionation, whilst highlighting the significant impact that operating conditions and membrane system selection have on processing.

## Supplementary Information

Below is the link to the electronic supplementary material.

ESM 1 (DOCX 2.16 MB)

## Data Availability

Data will be made available on request. This work was funded as part of a BBSRC CASE studentship (BB/T008776/1). This studentship is partly funded by Arla Foods Ingredients, a manufacturer with a commercial interest in increasing whey protein consumption. Arla Foods Ingredients were not involved in the evaluation or interpretation of results to ensure impartiality and took a supervisory reviewing role.
