# Supplementary material for: Optimising α-Lactalbumin Recovery from Whey via Membrane Filtration: The Role of Transmembrane Pressure Across Membranes Varying in Polymer Type and Pore Size
Source: Food Bioproc Tech. 2026 Feb 16;19(4):172. doi: 10.1007/s11947-026-04241-0 (PMC12909392; doi:10.1007/s11947-026-04241-0)
Supplement: Supplementary file 1 — (DOCX 2.16 MB) [file 11947_2026_4241_MOESM1_ESM.docx]

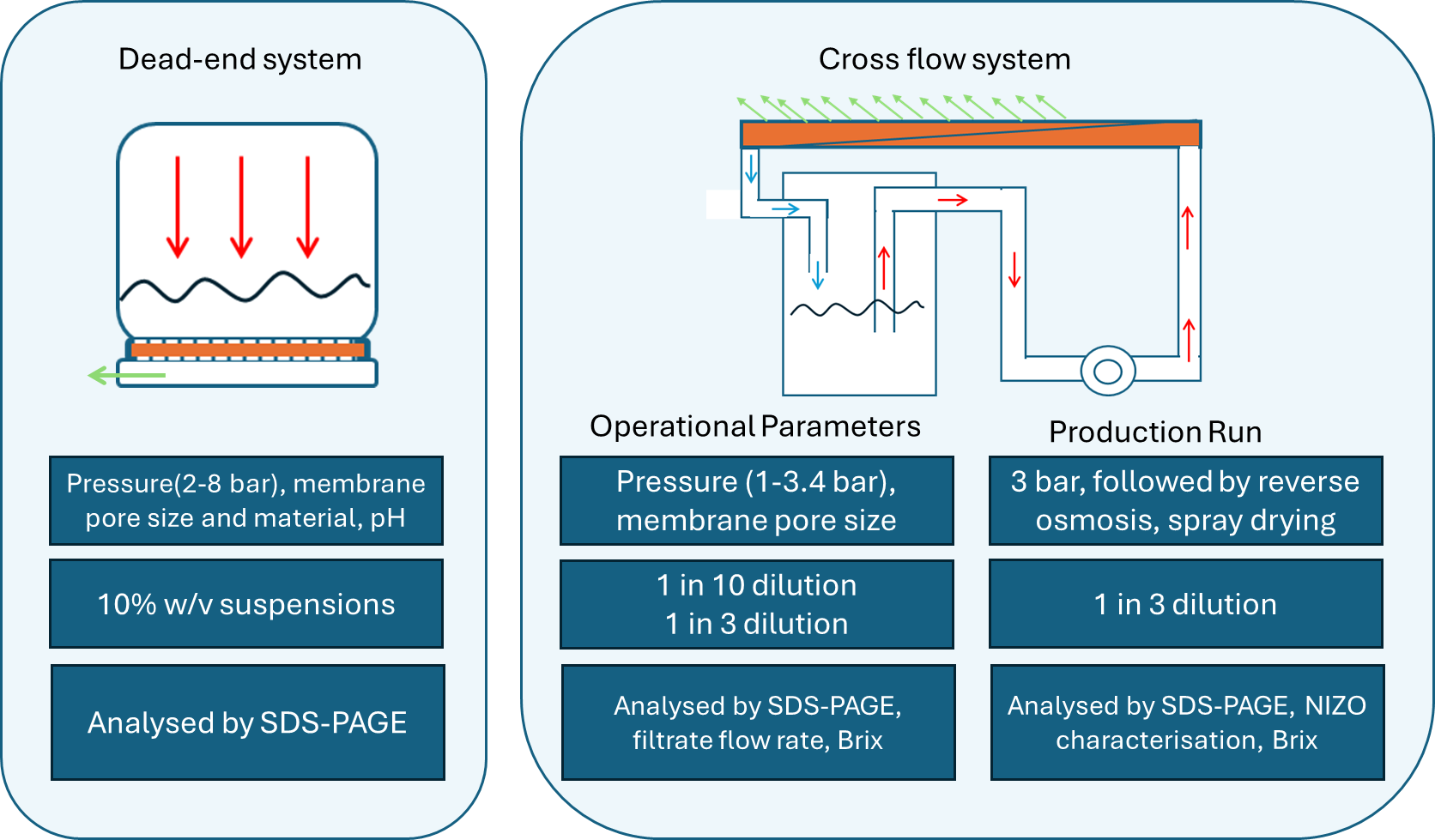
**7. Supplementary Figures**

*
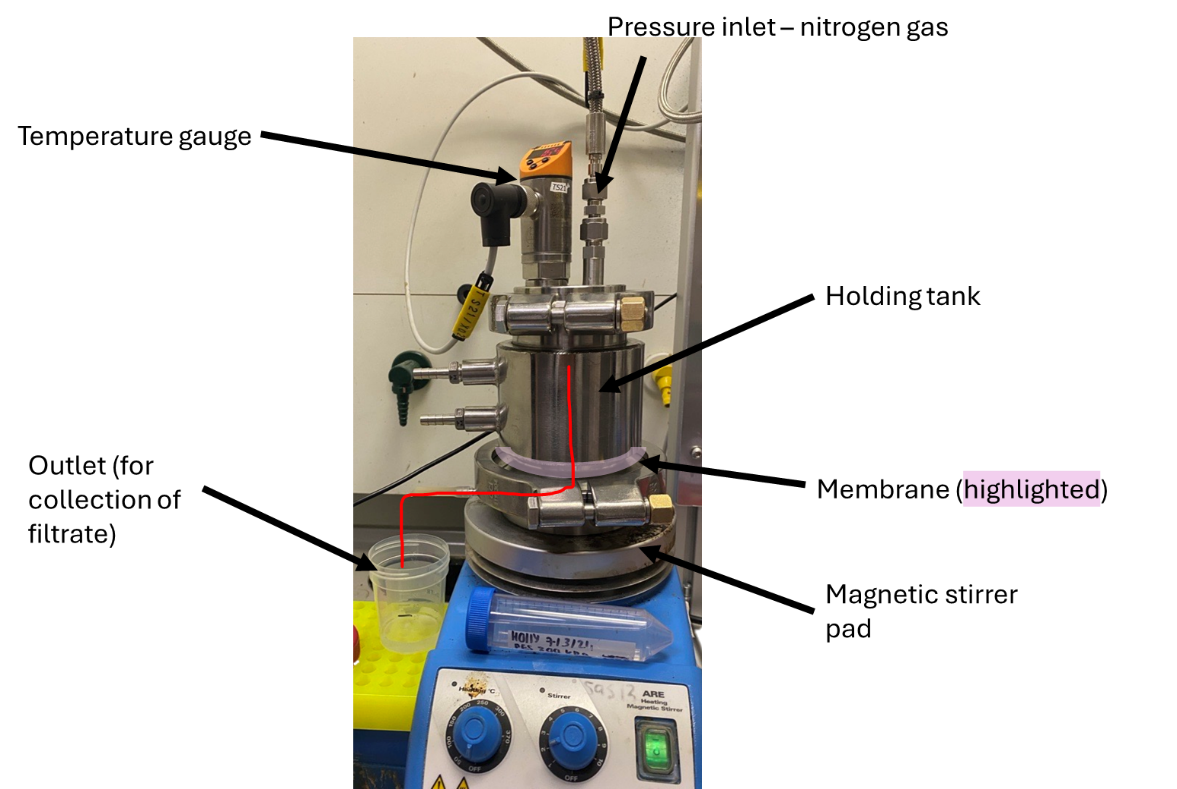
Supplementary Figure 1. Schematic showing an overview of the methodology with both the dead-end system and cross-flow system, including parameters investigated on each. Input of feed solution shown with red line: output of filtrate shown with green line, output of retentate into holding tank shown with blue line.*

*Supplementary Figure 2. Labelled image of the dead-end membrane filtration rig used in the study. Area containing membrane has been highlighted in pink. Flow of filtrate highlighted via red line.*

*
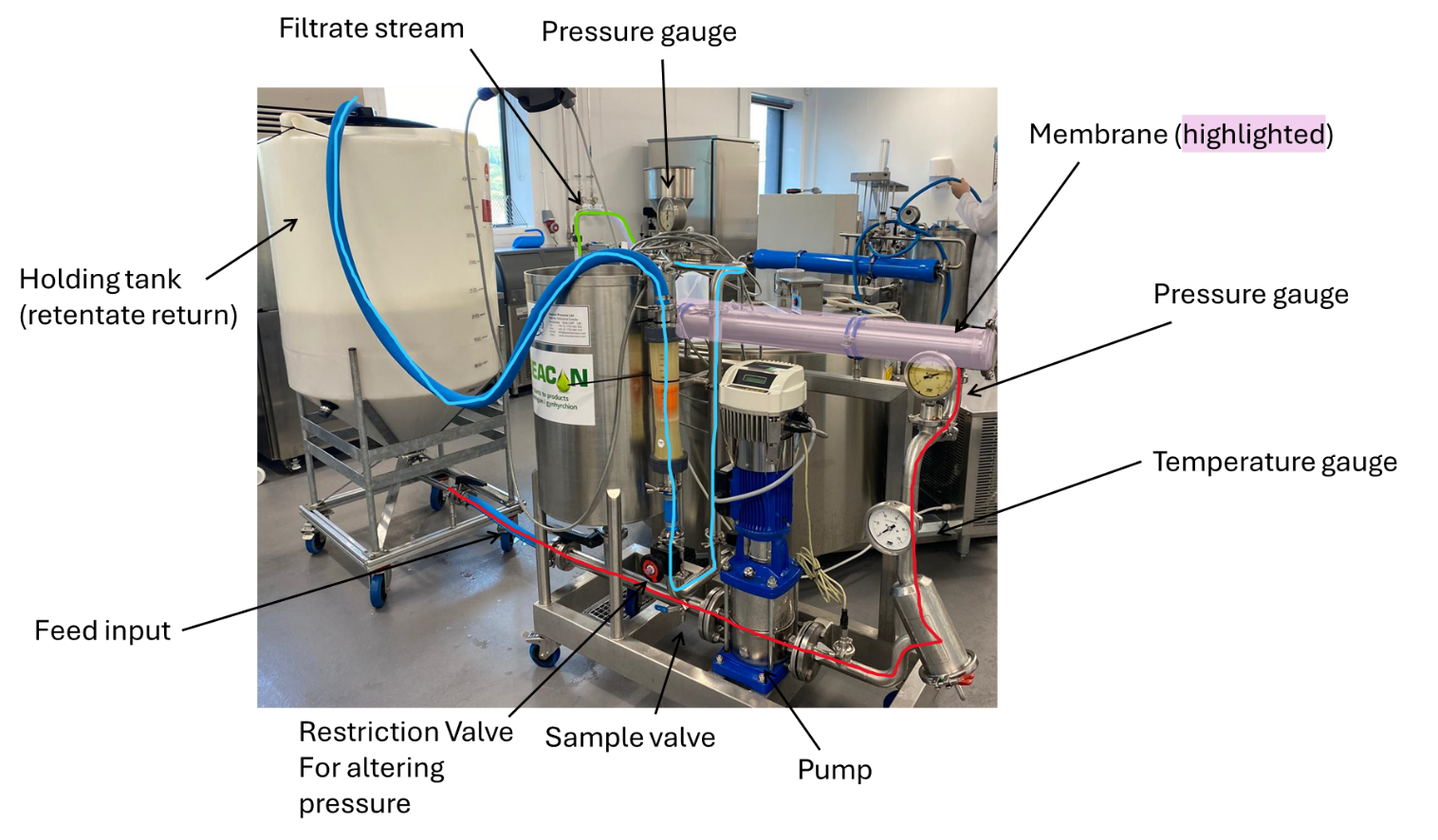
*

*
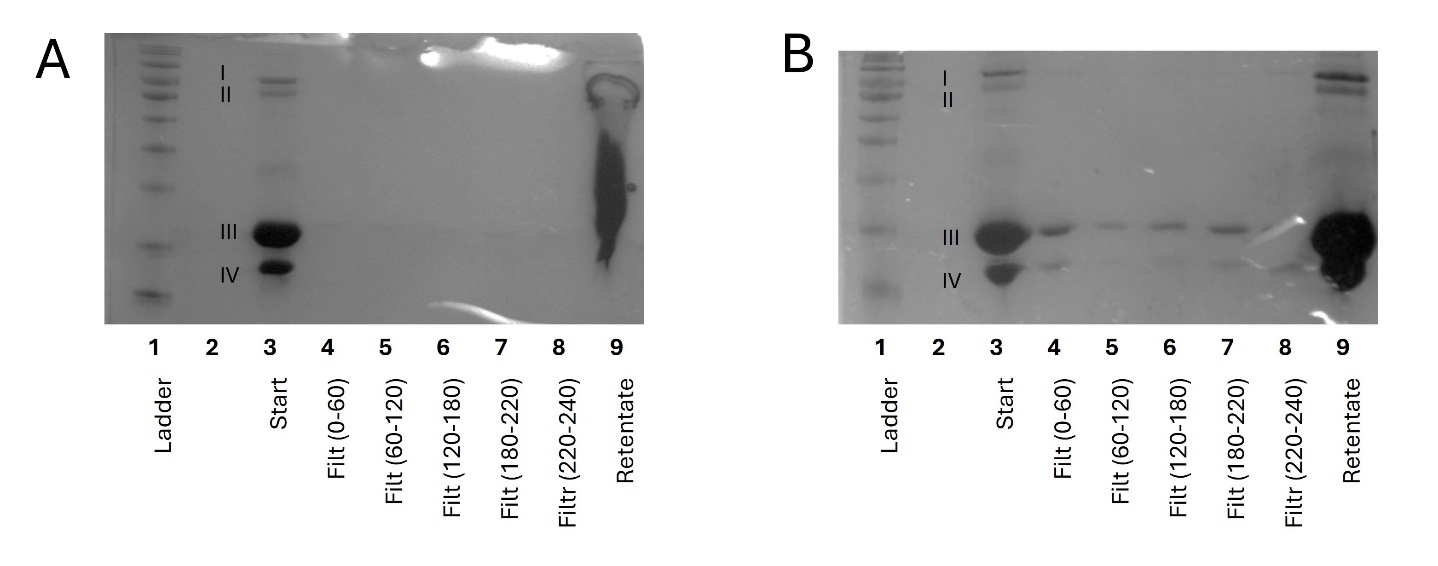
Supplementary Figure 3. Labelled image of the cross-flow membrane filtration rig used in the study. Area containing membrane has been highlighted in pink. Input of feed solution shown with red line: output of filtrate shown with green line, output of retentate into holding tank shown with blue line.*

*Supplementary Figure 4. SDS-PAGE of filtrates of 10% w/v whey protein isolate suspension using a commercially relevant ultrafiltration membrane operating at 8 bar with: [A] pH 3.2; [B] pH 6.5. Each well represents subsequent collections of filtrate, compared with a protein ladder, starting solution and retentate. Equal amounts loaded into each well (eg. not normalised by protein content).*
